# Development of a prediction model for bacteremia in hospitalized adults with cellulitis to aid in the efficient use of blood cultures: a retrospective cohort study

**DOI:** 10.1186/s12879-016-1907-2

**Published:** 2016-10-19

**Authors:** Chun-Yuan Lee, Calvin M. Kunin, Chung Chang, Susan Shin-Jung Lee, Yao-Shen Chen, Hung-Chin Tsai

**Affiliations:** 1 Division of Infectious Diseases, Department of Internal Medicine, Kaohsiung Medical University Hospital, Kaohsiung Medical University, Kaohsiung, Taiwan; 2 Department of Internal Medicine (CMK), Ohio State University, Columbus, OH USA; 3 Department of Internal Medicine (CMK), University of Arizona, Tucson, AZ USA; 4 Department of Applied Mathematics, National Sun Yat-sen University, Kaohsiung, Taiwan; 5 Division of Infectious Diseases, Department of Medicine, Kaohsiung Veterans General Hospital, Kaohsiung, Taiwan; 6 Faculty of Medicine, School of Medicine, National Yang-Ming University, Taipei, Taiwan; 7 Graduate Institute of Science Education and Environmental Education, National Kaohsiung Normal University, Kaohsiung, Taiwan

**Keywords:** Bacteremia, Cellulitis, Prediction model

## Abstract

**Background:**

Cellulitis is a common infectious disease. Although blood culture is frequently used in the diagnosis and subsequent treatment of cellulitis, it is a contentious diagnostic test. To help clinicians determine which patients should undergo blood culture for the management of cellulitis, a diagnostic scoring system referred to as the Bacteremia Score of Cellulitis was developed.

**Methods:**

Univariable and multivariable logistic regression analyses were performed as part of a retrospective cohort study of all adults diagnosed with cellulitis in a tertiary teaching hospital in Taiwan in 2013. Patients who underwent blood culture were used to develop a diagnostic prediction model where the main outcome measures were true bacteremia in cellulitis cases. Area under the receiver operating characteristics curve (AUC) was used to demonstrate the predictive power of the model, and bootstrapping was then used to validate the performance.

**Results:**

Three hundred fifty one cases with cellulitis who underwent blood culture were enrolled. The overall prevalence of true bacteremia was 33/351 cases (9.4 %). Multivariable logistic regression analysis showed optimal diagnostic discrimination for the combination of age ≥65 years (odds ratio [OR] = 3.9; 95 % confidence interval (CI), 1.5–10.1), involvement of non-lower extremities (OR = 4.0; 95 % CI, 1.5–10.6), liver cirrhosis (OR = 6.8; 95 % CI, 1.8–25.3), and systemic inflammatory response syndrome (SIRS) (OR = 15.2; 95 % CI, 4.8–48.0). These four independent factors were included in the initial formula, and the AUC for this combination of factors was 0.867 (95 % CI, 0.806–0.928). The rounded formula was 1 × (age ≥65 years) + 1.5 × (involvement of non-lower extremities) + 2 × (liver cirrhosis) + 2.5 × (SIRS). The overall prevalence of true bacteremia (9.4 %) in this study could be lowered to 1.0 % (low risk group, score ≤1.5) or raised to 14.7 % (medium risk group, score 2–3.5) and 41.2 % (high risk group, score ≥4.0), depending on different clinical scores.

**Conclusions:**

Determining the risk of bacteremia in patients with cellulitis will allow a more efficient use of blood cultures in the diagnosis and treatment of this condition. External validation of this preliminary scoring system in future trials is needed to optimize the test.

**Electronic supplementary material:**

The online version of this article (doi:10.1186/s12879-016-1907-2) contains supplementary material, which is available to authorized users.

## Background

Cellulitis is a common infectious disease which accounted for 10.1 % of infectious disease–related hospitalizations in US from 1998 to 2006 [1]. The infection can cause significant local tissue damage, and can spread systemically via the lymphatic system and bloodstream. The most common pathogens isolated from patients with non-purulent and purulent cellulitis are β-hemolytic streptococci and *Staphylococcus aureus*, respectively [2].

Guidelines for treating specific infectious diseases often emphasize the need to determine the microbial etiology of the invading pathogen(s) to plan the most effective antimicrobial therapy. However, in cases of cellulitis, diagnosis and management largely depend on the morphological features of the lesion and the clinical setting; the causal pathogen is less important [3]. The role of blood culture in the management of cellulitis remains especially controversial. This relates to the fact that the incidence of bacteremia in cellulitis is relatively low (2.0–18.5 %) [2, 4–9], and contamination rates are relatively high (3.6–4.1 %), which devalues the clinical significance of blood culture [4, 8]. Additionally, several studies suggest that blood culture is not cost-effective as a routine test, and has only a marginal effect on treatment efficacy [4, 8, 10, 11]. As a result, the 2014 Infectious Diseases Society of America (IDSA) guidelines for the management of skin and soft tissue infections state that blood cultures are recommended only for patients with deleterious conditions, such as malignancy, sepsis, immersion injury, animal bites, neutropenia, and severe cell-mediated immunodeficiency [3]. However, evidence to support these recommendations is limited.

Although there are many reported risk factors for bacteremia in cellulitis cases, including age >45 years, length of illness <2 days, temperature >38.5 °C on admission, a white blood cell count (WBC) >13,300/mm^3^ on admission, lymphedema, liver cirrhosis, and chronic kidney disease [4, 6, 9], they have not been subjected to multivariable analysis to more accurately identify cellulitis patients at risk of bacteremia, or risk stratification to quantify the probability of bacteremia in cellulitis patients. Therefore, when confronted with cellulitis, clinicians are often unable to discriminate which patients will benefit from blood culture examination immediately upon admission. This lack of quantified risk stratification and clear evidence-based guidance for cellulitis patients means that the majority of clinicians routinely order blood culture, even when there is a low risk of bacteremia [2, 4, 9]. This can be a waste of both time and medical resources, which is an increasingly important issue in the era of rising healthcare costs [12]. To identify the cellulitis patients who might benefit most from blood cultures, clinicians need an informative diagnostic scoring system to quantify a patient’s risk of bacteremia. With such a tool, the number of routine blood cultures performed in low risk groups might be significantly reduced. However, to the best of our knowledge, such a diagnostic prediction model to estimate the probability of true bacteremia in cellulitis has not yet been developed.

The aim of this study was to develop a diagnostic scoring system, referred to as the Bacteremia Score of Cellulitis, based on demographic data and clinical manifestations, to better predict cases of bacteremia in hospitalized adults. This system will provide a more evidence-based approach to order blood cultures, and could be implemented as part of clinical practice guidance for cellulitis.

## Methods

### Study design

To develop the multivariable prediction model for bacteremia in patients with cellulitis, all consecutive adult patients who were admitted to the Kaohsiung Veterans General Hospital (KVGH) with a diagnosis of cellulitis from January to December 2013 were retrospectively identified. KVGH is a tertiary teaching hospital in Taiwan. All available cases were used as part of this 1-year retrospective cohort study to maximize the power and generalizability of the results. The inclusion criteria were based on International Classification of Disease, Ninth Revision, Clinical Modification (ICD-9-CM) codes 528.3 (Cellulitis and abscess of oral soft tissues), 608.4 (Other inflammatory disorders of male genital organs), 681.00–681.9 (Cellulitis and abscess of finger and toe), and 682.0–682.9 (Other cellulitis and abscess) [2]. Patients aged ≤20 years, those found not to have cellulitis on chart review, and those with incomplete records (including patients without undergoing blood cultures) were excluded from the study. Enrolled patients were further subdivided into positive and negative blood culture groups (including results from sterile and contaminated cultures). These two groups were compared in terms of their demographic characteristics, underlying diseases, clinical manifestations, laboratory findings, and outcomes.

### Data collection

The computerized hospital records of all eligible patients were retrieved and reviewed. Candidate variables included all demographic, disease-related factors, clinical manifestations, and laboratory results on admission. Additionally, data related to blood culture, including the number of blood specimens used for culture, the number of positive cultures (if any), and the identification of all cultured isolates and their antimicrobial susceptibility, were also recorded. The primary outcome predicted by the diagnostic prediction model was true bacteremia, as defined below. All data except that related to blood culture was retrospectively recorded by one investigator who did not know the primary outcome of the study. Data related to blood culture was recorded by another investigator who had no knowledge of the results of the clinical data. Missing data were handled with complete case analysis, and cases with incomplete records were excluded from the study.

### Definitions

Some predictive variables were defined as follows. Wound and purulent and non-purulent cellulitis were defined as described previously [2, 13]. Involvement of non-lower extremities was defined as “involvement outside the lower extremities”. Patients were diagnosed with liver cirrhosis if coarse echogenicity, an irregular liver surface, and splenomegaly were detected upon abdominal sonography or computed tomography, or when signs of portal hypertension, such as esophageal varices or ascites, were noted in patients with chronic liver disease. Sepsis was defined according to the 2001 International Sepsis Definitions Conference as two or more of the following variables with suspicion of microbial processes: fever of more than 38 °C (100.4 °F) or body temperature less than 36 °C (96.8 °F); heart rate of more than 90 beats per min; respiratory rate of more than 20 breaths per min or arterial carbon dioxide tension of less than 32 mmHg; abnormal white blood cell count (>12,000/μL or <4000/μL, or >10 % immature [band] forms) [14].

The primary outcome, true bacteremia, was defined as positive blood cultures, excluding contaminants. Coagulase-negative staphylococci, diphtheroids, *Bacillus* species, and α-hemolytic streptococci were considered to be blood culture contaminants when they were isolated from only one culture bottle within a set, or when another set of blood culture bottles was sterile [15].

### Microbiological studies

Blood specimens were inoculated into resin-containing aerobic and anaerobic media (BACTEC Standard/10 Aerobic/F culture vials and BACTEC Standard Anaerobic/F culture vials, respectively) and incubated in a BACTEC 9240 System (Becton Dickinson, Sparks, MD, USA). Blood culture bottles were routinely incubated for up to 5 days. Terminal subcultures were not routinely performed unless clinically indicated. Bacteria were identified and antimicrobial susceptibility profiles were determined using a Vitek 2 automated system (bioMérieux, Saint Laurent, Canada) at the KVGH Clinical Microbiology Laboratory. All oxacillin-susceptible *S. aureus* isolates underwent confirmatory disk diffusion testing for cefoxitin susceptibility. All tests were performed and interpreted in accordance with Clinical and Laboratory Standards Institute guidelines (M100-S19) [16].

### Statistical analysis

Descriptive statistics were used to summarize the characteristics of patients with and without true bacteremia. In univariable analyses, the two groups were compared in terms of categorical and continuous variables using the chi-squared or Fisher’s exact tests and an independent *t*-test, respectively. All tests were two-tailed, with *p* < 0.05 considered significant. For the multivariable analysis of true bacteremia, candidate variables were identified as those having a univariate significance at *p* = 0.10, other variables identified in a published study, or those believed to be clinically meaningful. We considered variables with a multivariable *p*-value of <0.05 to be independent predictors of true bacteremia, and retained them to form the Bacteremia Score of Cellulitis formula. We assessed all second order interactions between the variables entered into the final multivariable logistic regression model. Interaction was considered to be present if the *p*-value associated with an interaction term was <0.05.

We quantified the ability of the model to discriminate between patients with and without true bacteremia by using the area under the receiver operating characteristics (ROC) curve (AUC), with 95 % confidence intervals (CI) [17]. Calibration plots were performed by comparing observed risk with predicted risk among seven subgroups of the full developmental model. Goodness of fit was quantified by using the Hosmer–Lemeshow test. To validate the results of the model coefficient and AUC, we randomly split the sample into an 80 % training set and a 20 % test set 1000 times i.e., the bootstrap method.

To further simplify the Bacteremia Score of Cellulitis, each logistic regression model coefficient was rounded to the nearest integer and used to assign weights (number of points) to each predictive factor. We used the final rounded model to estimate the probability of true bacteremia for each individual patient given a clinical score consisting of the sum of points. Clinical scores were further stratified into three risk groups (low, medium, and high) based on the probability of true bacteremia (placing cut points at the 25th and 75th percentiles of the model’s risk score distribution). All possible weighted combinations of a variable’s results led to different clinical scores, which could be used for decision making, depending on risk stratification.

We used SPSS version 22.0 (SPSS, Chicago, IL, USA) for all statistical analyses, except for internal validation. We used SAS version 9.3 (SAS Incorporated, Cary, NC, USA) to validate the model coefficient and the AUC of the developed model using resampling techniques (bootstrapping).

## Results

**Fig. 1:**
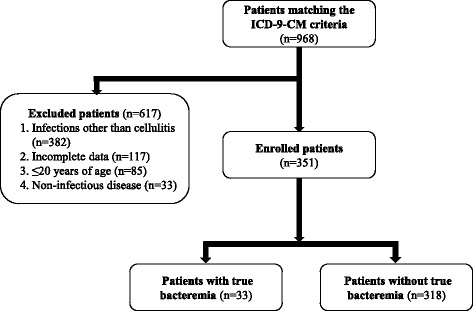
Flow of participants through the study

The participant flow diagram is outlined in Fig. 1. During the 1-year study period, 968 patients matching the ICD-9-CM criteria were hospitalized. Of these, 617 (63.7 %) were excluded following chart review. Of the 351 patients enrolled in this study, 33 (9.4 %) were positive blood culture group. There were a total of 691 blood samples (on average two cultures/patient). Contaminants, as defined in Methods, were isolated from a further 17 patients (4.8 %).

### Demographic characteristics and co-morbidities

Table 1 lists the demographic characteristics and co-morbidities of the 351 patients included in the study. The mean age was 62 ± 19.8 years, and 60.1 % were male. Compared with the negative blood culture group, the positive blood culture group had a higher rate of patients aged ≥65 years (63.6 % vs. 44.3 %; *p* = 0.034), and a higher incidence of liver cirrhosis (18.2 % vs. 6.0 %; *p* = 0.021). The two groups did not differ significantly with respect to sex, purulent features, co-morbidities (except liver cirrhosis), or duration of illness prior to admission following disease onset.

**Table 1:** Demographic characteristics and co-morbidities of the 351 cellulitis cases

	All (*n* = 351)	Positive blood culture (*n* = 33)	Negative blood culture (*n* = 318)	Odds ratio (95 % CI)	*p*-value
Mean (SD) age in years	62 (19.8)	68 (17.7)	61 (19.9)	N/A	0.055
Patients aged ≥65	162 (46.2)	21 (63.6)	141 (44.3)	2.20 (1.05–4.62)	0.034
Mean (SD) duration^a^ in days	4.5 (4.4)	3 (3.7)	4 (4.5)	N/A	0.155
Male sex	211 (60.1)	21 (63.6)	190 (59.7)	0.85 (0.40–1.79)	0.664
Wound	83 (23.6)	6 (18.2)	77 (24.2)	0.70 (0.28–1.75)	0.438
Purulent	60 (17.1)	9 (27.3)	51 (16.0)	1.96 (0.86–4.47)	0.103
Diabetes mellitus	95 (27.1)	14 (42.4)	81 (25.5)	2.16 (1.03–4.50)	0.37
Autoimmune disease	13 (3.7)	1 (3.0)	12 (3.8)	0.80 (0.10–6.33)	>0.999
Solid malignancy	55 (15.7)	9 (27.3)	46 (14.5)	2.22 (0.97–5.07)	0.54
Hematological malignancy	11 (3.1)	2 (6.1)	9 (2.8)	2.22 (0.46–10.71)	0.277
Liver cirrhosis	25 (7.1)	6 (18.2)	19 (6.0)	3.50 (1.29–9.49)	0.021
HIV infection	3 (0.9)	0 (0.0)	3 (0.9)	N/A	>0.999
Organ transplantation	2 (0.6)	1 (0.3)	1 (3.0)	9.91 (0.61–162.18)	0.179
Peripheral artery occlusive disease	10 (2.8)	1 (3.0)	9 (2.8)	1.07 (0.13–8.74)	>0.999
Coronary artery bypass graft	6 (1.7)	0 (0.0)	6 (1.9)	N/A	>0.999
Fracture	6 (1.7)	0 (0.0)	6 (1.9)	N/A	>0.999
Flap	8 (2.3)	1 (3.0)	7 (2.2)	1.39 (0.17–11.64)	0.550
Lymph node dissection	17 (4.8)	2 (6.1)	15 (4.7)	1.30 (0.29–5.97)	0.667

The data are expressed as number (%) unless otherwise indicated

*p*-values were determined by chi-squared or Fisher’s exact tests, or by independent *t*-test

*CI* confidence interval, *HIV* human immunodeficiency virus, *SD* standard deviation

^a^Duration following onset of disease, prior to admission

### Clinical signs, laboratory findings, and outcomes

The positive and negative blood culture groups differed significantly with respect to involvement of non-lower extremities (39.4 % vs. 19.2 %; *p* = 0.007) and SIRS on admission (81.8 % vs. 29.6 %; *p* < 0.001) (Table 2). The two groups did not differ significantly with respect to laboratory results and prescription of antimicrobial agent prior to blood culture.

**Table 2:** Clinical signs, laboratory findings, and outcomes of the 351 cellulitis cases

	All (*n* = 351)	Positive blood culture (*n* = 33)	Negative blood culture (*n* = 318)	Odds ratio (95 % CI)	*p*-value
Involvement of non-lower extremities	74 (21.1)	13 (39.4)	61 (19.2)	2.74 (1.29–5.82)	0.007
SIRS at admission	121 (34.5)	27 (81.8)	94 (29.6)	10.72 (4.29–26.82)	<0.001
WBC, 10^9^/L, mean (SD)	10.9 (5.1)	12.6 (6.5)	10.8 (4.9)	N/A	0.053
Lactic acid, mmol/L, mean (SD)	1.8 (1.2)	1.7 (0.9)	1.8 (1.2)	N/A	0.878
CRP, mg/dL, mean (SD)	7.3 (8.0)	9.8 (10.2)	7.0 (7.7)	N/A	0.149
GOT, IU/L, mean (SD)	33.2 (26.2)	36.3 (28.4)	32.9 (25.9)	N/A	0.544
GPT, IU/L, mean (SD)	31.6 (27.0)	33.8 (21.5)	31.4 (27.5)	N/A	0.616
Total bilirubin, mg/dL, mean (SD)	0.8 (1.0)	1.3 (1.8)	0.8 (0.7)	N/A	0.148
Prescription of antimicrobial agent prior to blood culture	7 (1.99)	1 (3.03)	6 (1.89)	1.63 (0.19–13.92)	0.655

The data are expressed as number (%) unless otherwise indicated

*p*-values were determined by chi-squared or Fisher’s exact tests, or by independent *t*-test

*CI* confidence interval, *CRP* C-reactive protein, *GOT* glutamate oxaloacetate transaminase, *GPT* glutamate pyruvate transaminase, *SIRS* systemic inflammatory response syndrome, *SD* standard deviation, *WBC* white blood cell count

### Risk factors and risk stratification for true bacteremia

Multivariable logistic regression analysis showed that the risk of true bacteremia was higher in older patients (≥65 years) (odds ratio [OR] = 3.9; 95 % CI, 1.5–10.1; *p* = 0.05), when non-lower extremities were involved (OR = 4.0; 95 % CI, 1.5–10.6; *p* = 0.05), and when there was liver cirrhosis (OR = 6.8; 95 % CI, 1.8–25.3; *p* = 0.05) or SIRS (OR = 15.2; 95 % CI, 4.8–48.0; *p* < 0.001) (Table 3).

**Table 3:** Multivariable analysis to identify the risk factors for true bacteremia in the 351 cellulitis cases

Variables	OR	95 % CI	*p-*value
Age ≥65 years	3.924	1.524–10.104	0.05
Duration after onset of disease, prior to admission (days)	0.998	0.885–1.125	0.977
Purulent group	1.899	0.629–5.734	0.256
Liver cirrhosis	6.760	1.805–25.316	0.05
SIRS	15.182	4.802–47.997	<0.001
Involvement of non-lower extremities	3.994	1.504–10.603	0.05
WBC	0.948	0.874–1.029	0.204
CRP	1.006	0.954–1.060	0.836

*CI* confidence interval, *CRP* C-reactive protein, *OR* odds ratio, *SIRS* systemic inflammatory response syndrome, *WBC* white blood cell count

**Fig. 2:**
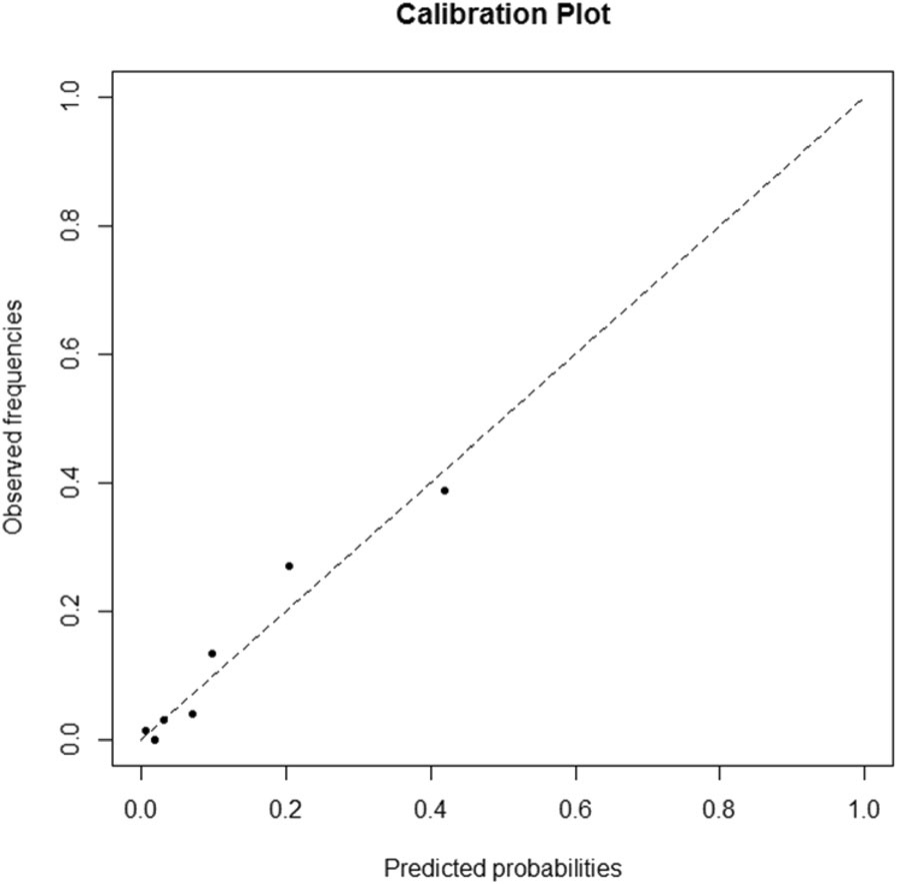
Calibration plot of observed versus predicted risk using the full developmental model (*n* = 351)

These four predictors were retained in the multivariable regression analysis, and no statistical interactions were noted. Table 4 lists the ORs of these four independent predictors and their clinical scores. The logistic regression model coefficients of the four independent variables were used to develop the Bacteremia Score of Cellulitis formula (Table 4). The initial Bacteremia Score of Cellulitis formula was: 1.138 × (age ≥65 years) + 1.624 × (non-lower extremities involved) + 2.161 × (liver cirrhosis) + 2.601 × (SIRS). The discriminatory power of this initial score, as assessed by the AUC, was 0.867 (95 % CI, 0.806–0.928). The calibration plot of observed risk versus predicted risk using the full developmental model is provided in Fig. 2, and the Hosmer–Lemeshow goodness of fit test had a *p*-value of 0.472, indicating that the model does not misrepresent the data. Internal validation of the model coefficient and AUC by bootstrapping showed little indication of undue influence by particular patients in the training set (Additional file 1).

**Table 4:** Calculation of the Bacteremia Score of Cellulitis on the basis of the variables identified by the logistic regression model and the discriminatory power assessed by the area under the ROC curve

Variable	Odds ratio (95 % CI)	Coefficient (β)	*p-*value	Clinical score^a^
Age ≥65 years	3.12 (1.31–7.42)	1.138	0.010	1
Involvement of non-lower limb extremities	5.08 (2.06–12.51)	1.624	<0.001	1.5
Liver cirrhosis	8.68 (2.52–29.88)	2.161	0.001	2
SIRS	13.48 (4.99–36.42)	2.601	<0.001	2.5
Area under ROC curve (95 % CI)				
	Initial Bacteremia Score of Cellulitis^b^	0.867 (0.806–0.928)		
	Rounded Bacteremia Score of Cellulitis^c^	0.865 (0.804–0.926)		

*ROC* receiver operating characteristics, *CI* confidence interval; SIRS, systemic inflammatory response syndrome

^a^Clinical scores of each variable present are added. For example, a patient aged ≥65 years, involvement of non-lower limb extremities, SIRS, and no history of liver cirrhosis has a clinical score of: 1 + 1.5 + 0 + 2.5 = 5.0

^b^Initial Bacteremia Score of Cellulitis = 1.138 × age ≥65 years + 1.624 × involvement of non-lower extremities + 2.161 × liver cirrhosis + 2.601 × SIRS status

^c^Rounded Bacteremia Score of Cellulitis = 1 × age ≥65 + 1.5 × involvement of non-lower extremities + 2 × liver cirrhosis + 2.5 × SIRS status

**Fig. 3:**
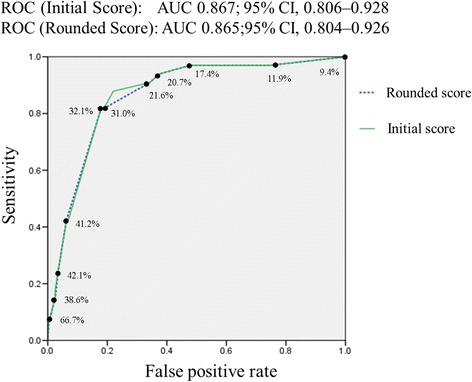
Comparison of ROC of both initial and rounded score with predicted risks labelled on the curve

**Fig. 4:**
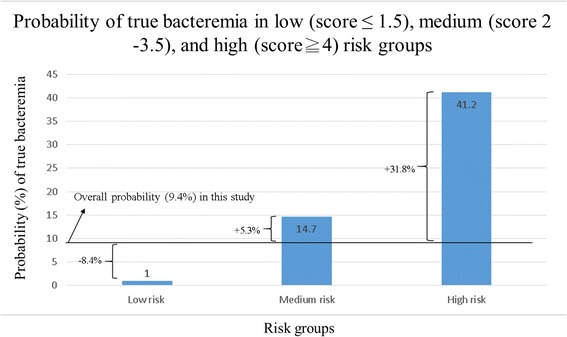
Risk stratification of the Bacteremia Score of Cellulitis based on the probability of true bacteremia, and the difference in the probability of true bacteremia between the overall study population and each risk group. Overall prevalence of positive culture = 9.4 % (33/351). The probability of true bacteremia in the low (score ≤1.5), medium (score 2.0–3.5), and high (score ≥4.0) risk groups was 1.0 %, 14.7 %, and 41.2 %, respectively. The difference in probability between the overall study population and the low, medium, and high risk groups was −8.4 %, 5.3 %, and 31.8 %, respectively

The integer-based rounded Bacteremia Score of Cellulitis formula was 1 × (age ≥65 years) + 1.5 × (involvement of non-lower extremities) + 2 × (liver cirrhosis) + 2.5 × (SIRS). The discriminatory power of this rounded score, as assessed by the AUC, was 0.865 (95 % CI, 0.804–0.926) (Fig. 3). The rounded formula generated 16 different combinations of test results. These combinations corresponded to 13 different clinical scores, varying from 0 to 7 (Table 5). Clinical scores were then stratified into low (score ≤1.5), medium (score 2–3.5), and high (score ≥4) risk groups. For a patient belonging to the low risk group, the probability of true bacteremia decreased from 9.4 % (overall prevalence in this study) to 1.0 %, and applied to the majority (57.5 %) of patients. By contrast, belonging to the high risk group raised the probability of true bacteremia to 41.2 % (Fig. 4). When the cut-off value of 2.0 was used, indicating that only patients with a clinical score of 2 or higher should receive blood culture, the rounded Bacteremia Score of Cellulitis had a sensitivity, specificity, positive predictive value, and negative predictive value of 93.9 %, 62.6 %, 20.7 %, and 99.0 %, respectively.

**Table 5:** Clinical scores and their associated probabilities of true bacteremia, sensitivity, and specificity

Clinical score	Probabilities of true bacteremia (%)^a^	Percentage correctly having blood culture(s) (sensitivity)^b^	Percentage correctly rejecting blood culture(s) (specificity)^c^
7.0	N/A	0	100
6.0	N/A	0	100
5.5	66.7 (2/3)	6.1	99.7
5.0	38.6 (5/13)	15.2	97.5
4.5	42.1 (8/19)	24.2	96.5
4.0	41.2 (14/34)	42.4	93.7
3.5	32.1 (27/84)	81.8	82.1
3.0	31.0 (27/87)	81.8	81.1
2.5	21.6 (30/139)	90.9	65.7
2.0^d^	20.7 (31/150)	93.9	62.6
1.5	17.4 (32/184)	97	52.2
1.0	11.9 (32/269)	97	25.5
0	9.4 (33/351)	100	0

^a^Indicates the probabilities of true bacteremia in cellulitis cases using the clinical score in that row as a cut-off point to determine whether to order a blood culture. In parenthesis are the number of cellulitis cases with true bacteremia (numerator) and total number of cellulitis cases (denominator) using the clinical score in that row as a cut-off point

^b^Fraction of patients with true bacteremia who would correctly have blood culture(s) if the clinical score in that row was used as a cut-off point

^c^Fraction of patients without true bacteremia who would correctly reject blood culture(s) if the clinical score in that row was used as a cut-off point

^d^Clinical score of 2.0 was used to illustrate its use for decision-making

### Microbiological results

The pathogens isolated from these 33 cases are listed in Table 6. β-hemolytic streptococci were isolated from 16 patients (48.5 %) (group G *Streptococcus*, *n* = 8; group B *Streptococcus*, *n* = 5; group A *Streptococcus*, *n* = 3). *S. aureus* was isolated from seven (21.2 %) patients (methicillin-resistant *S. aureus*, *n* = 4; methicillin-susceptible *S. aureus*, *n* = 3). Gram-negative bacilli (GNB) were isolated from eight (24.2 %) patients.

**Table 6:** Pathogens isolated from blood culture in the 33 cases of true bacteremia

Organism	No. (%) of positive cultures
Group G *Streptococcus*	8 (24.2)
Group B *Streptococcus*	5 (15.2)
Methicillin-resistant *Staphylococcus aureus*	4 (12.1)
Methicillin-susceptible *Staphylococcu*s *aureus*	3 (9.1)
Group A *Streptococcus*	3 (9.1)
*Staphylococcus lugdunensis*	2 (6.1)
*Klebsiella pneumoniae*	2 (6.1)
*Klebsiella pneumoniae* and *Salmonella* serotype group D	1 (3.0)
*Acinetobacter junii*	1 (3.0)
*Enterobacter cloacae* complex	1 (3.0)
*Morganella morganii*	1 (3.0)
*Pseudomonas aeruginosa*	1 (3.0)
*Salmonella*	1 (3.0)

Data are expressed as number (%)

## Discussion

This analysis was used to develop an initial diagnostic prediction model that might help primary physicians in the decision-making process surrounding initial diagnosis of bacteremia in cases of cellulitis. Although the rounded score performed well in predicting bacteremia in our study population (AUC = 0.865; 95 % CI, 0.804–0.926), it still needs further validation with independent samples before it can be used in clinical care.

Our findings showed that the weighted combination of four independent predictors (age ≥65 years, involvement of non-lower extremities, liver cirrhosis, and SIRS) can help discriminate between patients with and without true bacteremia in hospitalized adults with cellulitis. This Bacteremia Score of Cellulitis employs clinical and demographic variables to stratify cellulitis patients, and thereby identify those at higher risk of true bacteremia. The medium and high risk groups were more likely to benefit from blood culture than the overall population in this study (14.7 % and 41.2 % vs. 9.4 %, respectively). Conversely, the study patients who had a rounded Bacteremia Score of Cellulitis of ≤1.5 were at much lower risk of true bacteremia than the overall population (1.0 % vs. 9.4 %) (Fig. 4). The Bacteremia Score of Cellulitis will thus be useful for stratifying hospitalized adults with cellulitis according to their bacteremia risk, which in turn will help clinicians optimize decision making on when to order blood cultures on admission.

However, without more cost-effective decision analyses, the choice of a so-called rational cut-off value to determine whether to order a blood culture seems somewhat arbitrary. When we used a cut-off value of 2.0, the majority (57.5 %) of patients were stratified into the low risk group. With a high negative predictive value (99.0 % in the present study), a cut-off value of 2 is a good negative screening threshold, thereby reducing the number of routine blood cultures in low risk groups. This could lead to a substantial reduction in the costs associated with blood cultures. However, the use of a cut-off value means that some patients with true bacteremia will not be identified. In the current study, two cases of true bacteremia in the low risk group would not be identified if a cut-off value of 2 was used. In one 54-year-old man without co-morbidities, the antimicrobial treatment was de-escalated from vancomycin to penicillin based on isolation of group B *Streptococcus* from blood culture. In the other, a 50-year-old man with history of allograft stem cell transplantation for acute myeloid leukemia, the antimicrobial treatment was switched from oxacillin to piperacillin according to isolation of *Morganella morganii*. The role of immunocompromised status (i.e., patients receiving immunosuppressive agents or chemotherapy, those with hematological malignancy, or those infected with human immunodeficiency virus) in this prediction model remains unknown because of the small number of immunocompromised patients in our study. However, even without further evidence, it is reasonable to assume that blood cultures would be beneficial for cellulitis patients with deleterious conditions such as malignancy, neutropenia, and severe cell-mediated immunodeficiency [3], even in cases with a rounded Bacteremia Score of Cellulitis ≤1.5.

The present study showed that cirrhosis was a predictor for true bacteremia in cellulitis cases. This may reflect the multiple immune system defects associated with cirrhosis, including reduced polymorphonuclear leukocyte activity, reduced Kupffer cell numbers, and increased bacterial translocation from the gut resulting from altered intestinal immunity and bacterial overgrowth [18]. This immune dysfunction may promote bacteremia caused by GNB, leading to hematogenous seeding to the skin and soft tissue at distant sites [18].

The present study identified involvement of non-lower extremities as an independent predictor of bacteremia in cellulitis, which has not been reported previously. In most cases, cellulitis is caused by a microbial breach of the cutaneous layer, particularly in patients with predisposing conditions [3]. Such infections are most common in the lower limbs [3]. For cellulitis localized outside the bilateral lower limbs, we hypothesize that the condition may be caused by hematogenous seeding of bacteria into the skin and soft tissue from a more distant site, rather than a local microbial breach. This may explain why the involvement of non-lower extremities is a predictor for bacteremia in cellulitis.

Consistent with previous studies [7], we found that β-hemolytic streptococci were the most common bacteria isolated from the 33 positive blood cultures (*n* = 16; 48.5 %). Moreover, non-group A β-hemolytic streptococci were isolated more frequently than group A β-hemolytic streptococci (39.4 % vs. 9.1 %). Group G *Streptococcus* was the most commonly isolated pathogen (24.2 %), which confirms the findings of other studies [9, 19]. However, a notable finding of the present study was that GNB were isolated in 24.2 % (8/33) of bacteremia cases. The 2014 IDSA guidelines do not recommend empirical antimicrobial therapy against GNB for the management of erysipelas and cellulitis except in severely compromised patients, in whom broad-spectrum antimicrobial agents such as vancomycin plus either piperacillin-tazobactam or imipenem/meropenem are recommended [3]. Because we only identified 33 cases of true bacteremia in the current study, further multicenter studies are needed to identify the risk factors for GNB bacteremia in cellulitis.

Our study design has several limitations. First, because it is retrospective in nature, clinical signs and co-morbidities were only derived from patient records. Second, although the prescription rate for antimicrobial agents prior to blood culture was not significantly different between the two groups (negative blood culture vs. positive blood culture: 1.89 % vs. 3.03 %, *p* = 0.66), it was difficult to accurately determine the true timing of prescription of antimicrobial agents relative to blood culture sampling from the computerized records. Further prospective studies are needed to control the confounding factor of pretreatment with antimicrobial agent. Third, the prediction model was developed for hospitalized patients in a tertiary teaching hospital in Taiwan over a 1-year period. Thus, the selection bias of a population with a higher degree of disease severity is inevitable, and our findings may not be generalizable to other hospital or outpatient settings in Taiwan or overseas. In addition, the study cohort seems to be limited for model training and testing as performed in our study. Therefore, external validation with an independent sample, either from a different time period at the same collection site or from a different collection site, is needed to validate our preliminary results and evaluate the generalizability of the model. Finally, this study was limited to adults. Because of differences in demography, disease co-morbidities, and microbiology, the results of the current study cannot be applied to children [20].

## Conclusions

This study has provided a preliminary, diagnostic prediction model to estimate the probability of true bacteremia in cases of cellulitis. Based on the demographic and clinical characteristics of patients upon admission, the Bacteremia Score of Cellulitis may provide a simple prediction model to stratify cellulitis patients into low, medium, and high risk groups, and help clinicians optimize decision making upon admission. However, this prediction model has several limitations, as described above. A multicenter prospective study with a larger number of cellulitis patients, including more immunocompromised hosts and children, is needed to validate the Bacteremia Score of Cellulitis. This may lead to the development of an easy-to-use and validated diagnostic model to restrict the drawing of blood cultures in cellulitis cases to those with a high probability of true bacteremia.

## Additional file

Additional file 1: Internal validation of model coefficient and AUC in training set by bootstrapping. (PPT 1084 kb)

## Abbreviations

AUC: Area under the receiver operating characteristics curve
CI: Confidence interval
CRP: C-reactive protein
GNB: Gram-negative bacilli
GOT: Glutamate oxaloacetate transaminase
GPT: Glutamate pyruvate transaminase
HIV: Human immunodeficiency virus
ICD-9-CM: International classification of disease, ninth revision, clinical modification
IDSA: Infectious Diseases Society of America
KVGH: Kaohsiung Veterans General Hospital
OR: Odds ratio
ROC: Receiver operating characteristics
SD: Standard deviation
SIRS: Systemic inflammatory response syndrome
WBC: White blood cell count
