# Supplementary material for: Development of a prediction model for bacteremia in hospitalized adults with cellulitis to aid in the efficient use of blood cultures: a retrospective cohort study
Source: BMC Infect Dis. 2016 Oct 19;16:581. doi: 10.1186/s12879-016-1907-2 (PMC5070006; doi:10.1186/s12879-016-1907-2)

## Slide 1
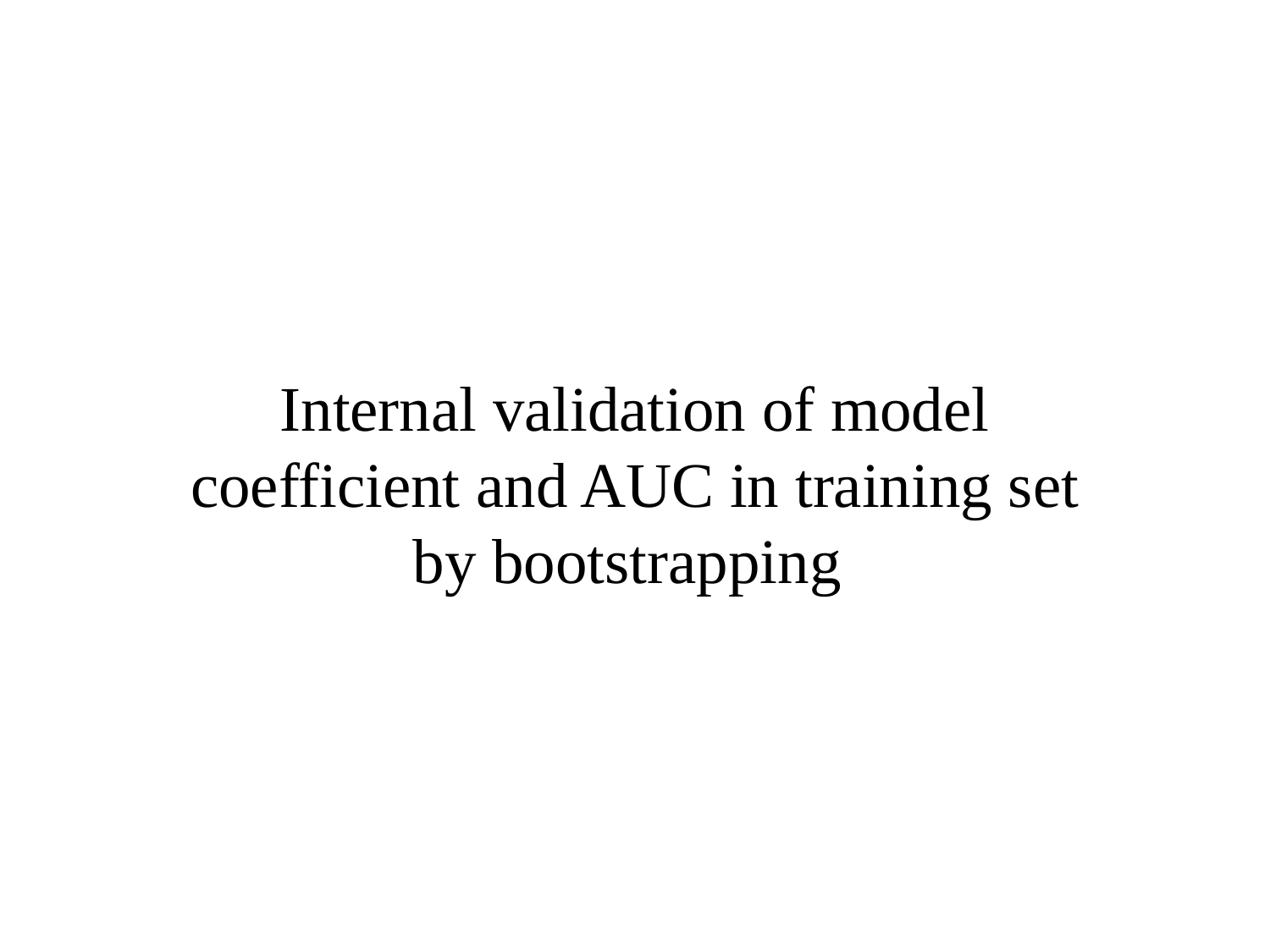

# Internal validation of model coefficient and AUC in training set by bootstrapping

## Slide 2
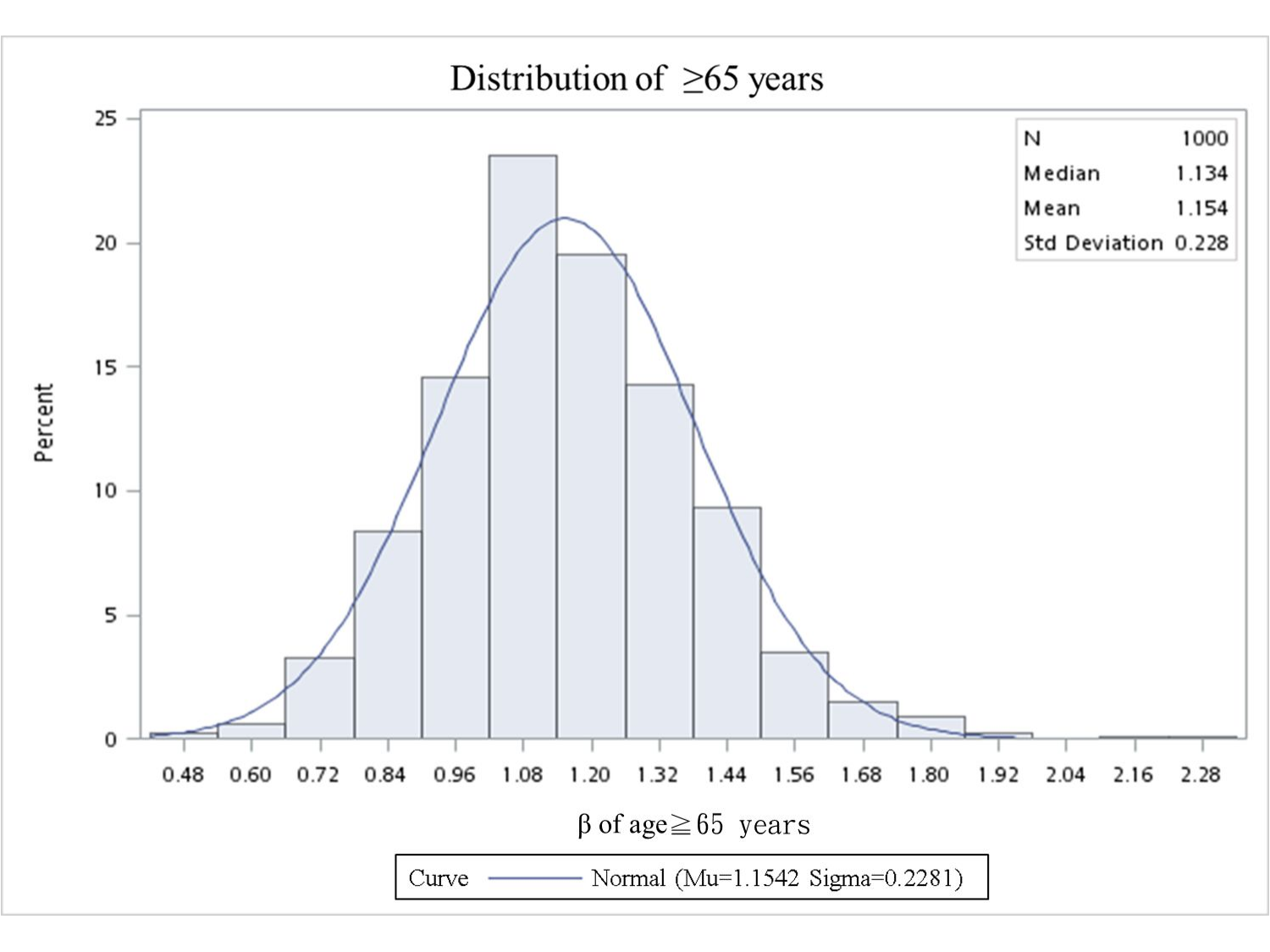

## Slide 3
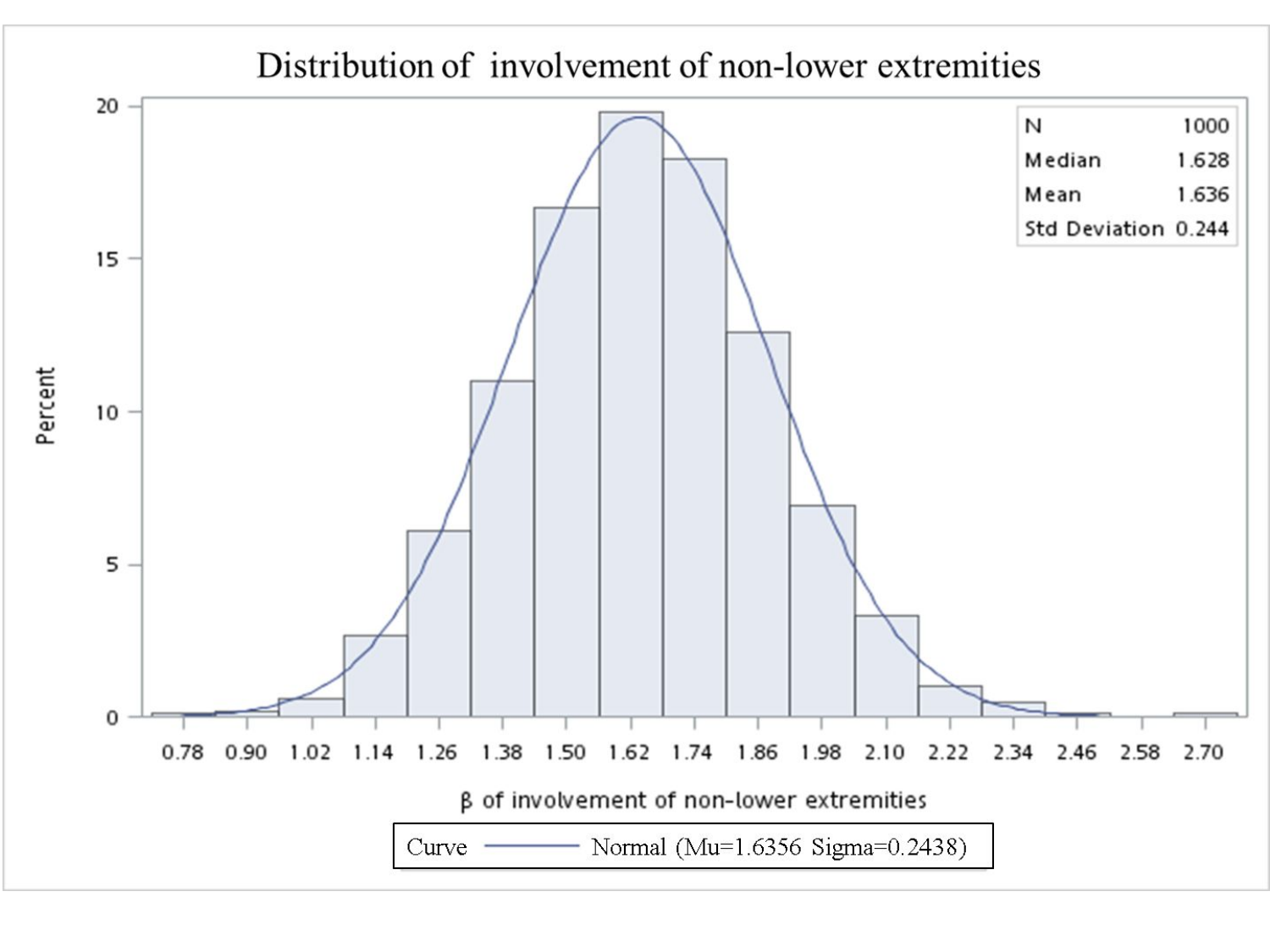

## Slide 4
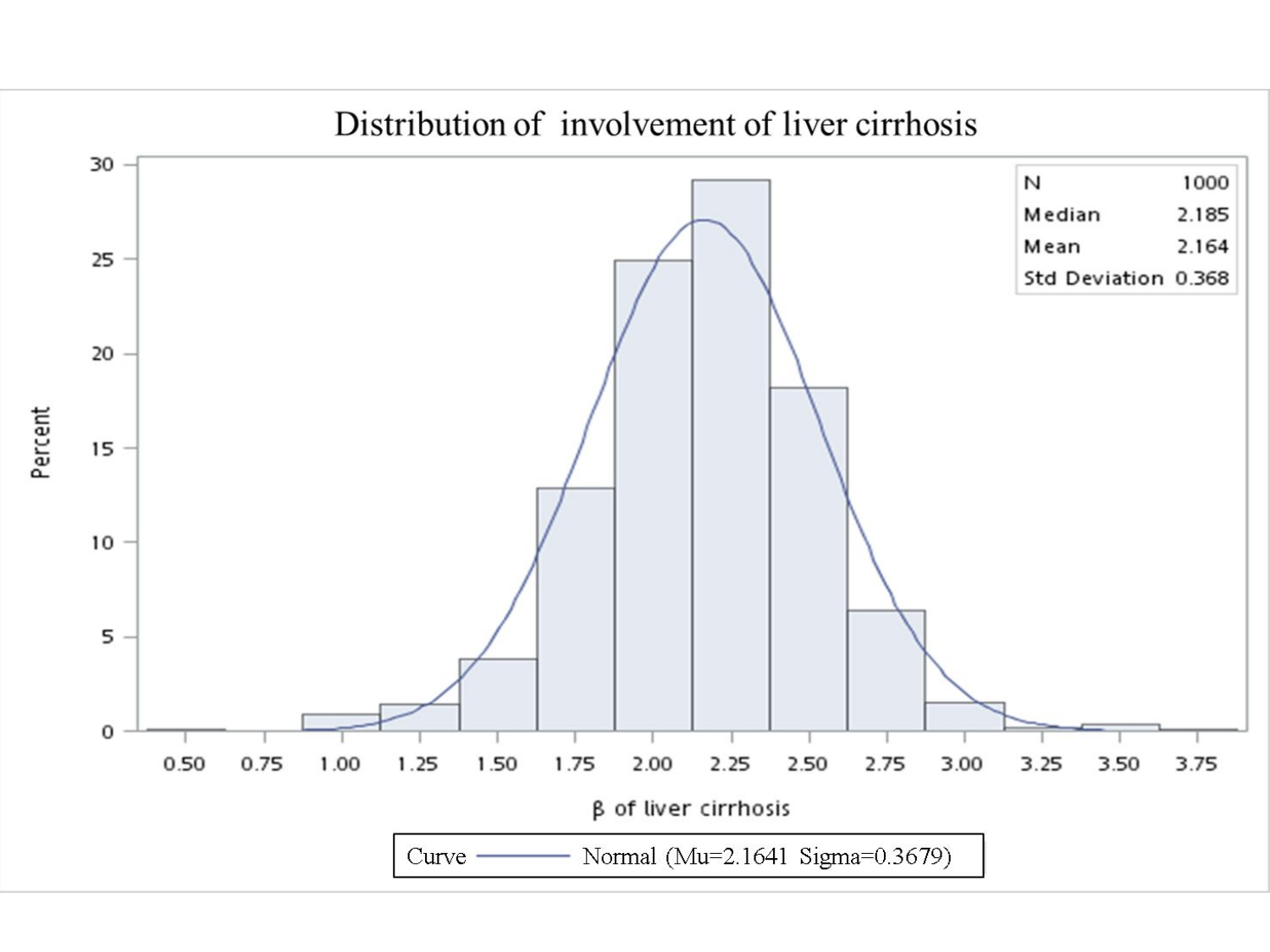

## Slide 5
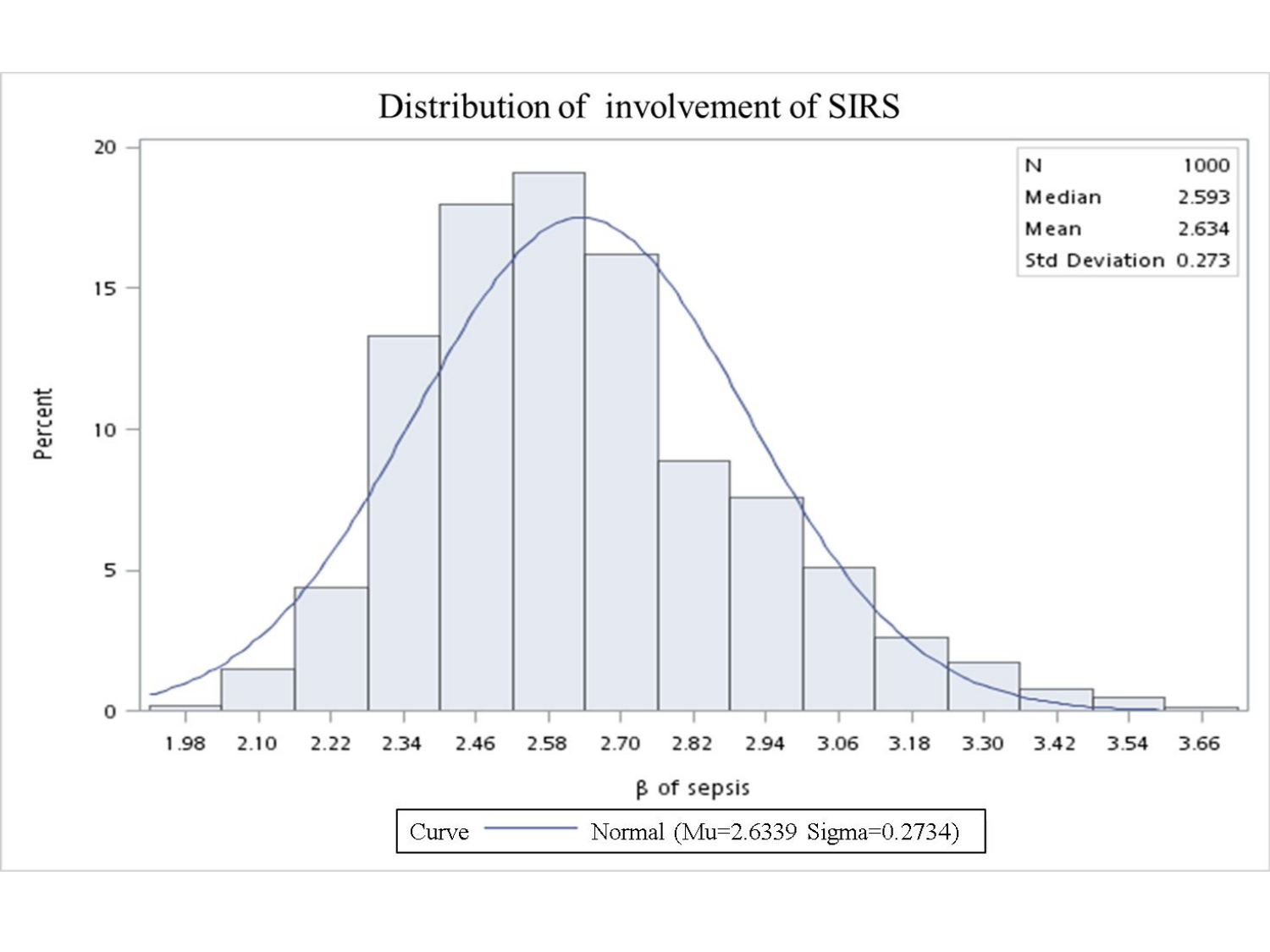

## Slide 6
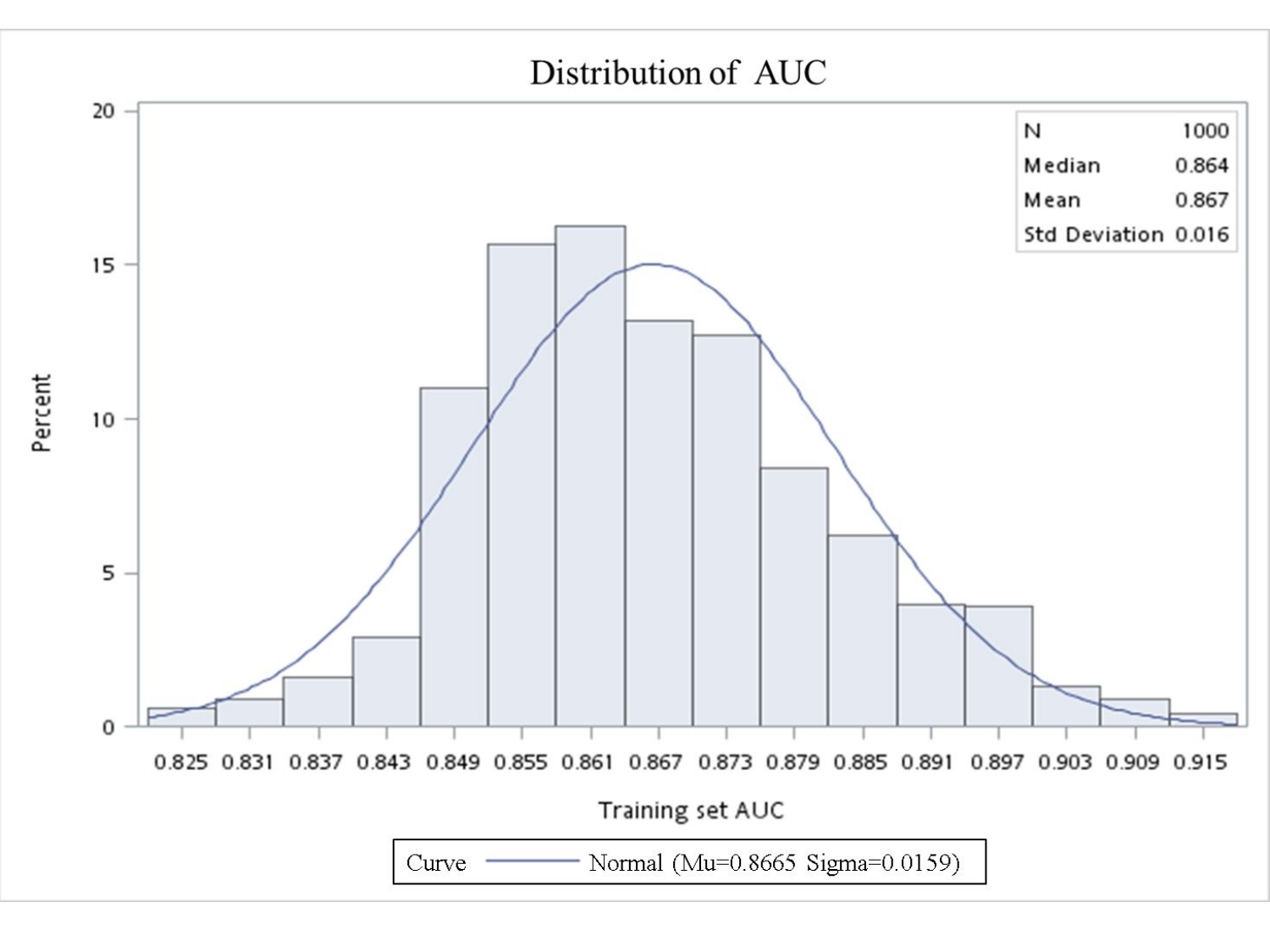

Supplement: Additional file 1: — Internal validation of model coefficient and AUC in training set by bootstrapping. (PPT 1084 kb) [file 12879_2016_1907_MOESM1_ESM.ppt]
